# Left-Sided Whole Breast Irradiation with Hybrid-IMRT and Helical Tomotherapy Dosimetric Comparison

**DOI:** 10.1155/2014/741326

**Published:** 2014-07-13

**Authors:** An-Cheng Shiau, Chen-Hsi Hsieh, Hui-Ju Tien, Hsin-Pei Yeh, Chi-Ta Lin, Pei-Wei Shueng, Le-Jung Wu

**Affiliations:** ^1^Division of Radiation Oncology, Department of Radiology, Far Eastern Memorial Hospital, Section 2, Nan-Ya South Road, Banqiao, New Taipei City 220, Taiwan; ^2^Department of Biomedical Imaging and Radiological Science, China Medical University, Taichung 404, Taiwan; ^3^Department of Biomedical Imaging and Radiological Sciences, National Yang-Ming University, Taipei 112, Taiwan; ^4^Department of Radiation Oncology, Tri-Service General Hospital, National Defense Medical Center, Taipei 114, Taiwan

## Abstract

*Purpose*. Limited-tomotherapy and hybrid-IMRT treatment techniques were compared for reductions in ipsilateral and contralateral lung, heart, and contralateral breast radiation doses.* Methods and Materials.* Thirty consecutively treated left-sided early-stage breast cancer patients were scheduled for lTomo and hIMRT. For the hIMRT plan conventional tangential-field and four-field IMRT plans were combined with different weightings in the prescribed dose. For the lTomo plan a geometrically limited arc was designed for the beamlet entrance. A *D*_*p*_ of 50.4 Gy in 28 fractions was used for the PTV. The dose coverage, homogeneity index, conformity index of the target, and the dose volumes of critical structures were compared.* Results.* Both modalities presented similar target coverage. The homogeneity and conformity were improved for lTomo with *P* < 0.001 and *P* = 0.006, respectively. In the lTomo plan a concave dose distribution was generated with significant dose reductions in both high and low dose regions for ipsilateral lung and heart (*P* < 0.001).* Conclusions*. lTomo plan can have similar dose coverage and better homogeneity and conformity to the target. By properly designing the directionally and completely blocked structure, lTomo plan was developed successfully in reducing doses to the healthy tissues for early-stage left-sided breast cancer radiotherapy.

## 1. Introduction

Radiotherapy is an integral treatment after breast conserving surgery for patients with early-stage breast cancer. Radiotherapy can reduce the rates of recurrence and death from breast cancer [1]. Treatment planning typically involves delivering an adequate dose to the intact breast while minimizing doses to the critical lung, heart, and contralateral breast organs using the conventional tangential field technique. However, when treating left-sided early-stage breast cancer the concave shape of the target results in a high dose of irradiation to parts of the ipsilateral lung and heart with tangential fields. Additionally the high dose region is located at the anterior heart, including the left anterior descending (LAD) coronary artery, causing increases in perfusion defects after radiation treatment [2, 3]. Long-tern follow-up of these patients has revealed that radiation therapy can also increase the risk for ischemic heart disease [1, 4–7]. A recent study showed that the risk for major coronary events (i.e., myocardial infraction, coronary revascularization, or death from ischemic heart disease) increased linearly with the mean dose to the heart, with no apparent threshold [6]. Doses to the heart from radiotherapy for left-sided breast cancer vary widely. The greatest source of variability occurs in doses from the distance of the heart to the thoracic wall, with the mean dose about 0.9–15 Gy [4, 8, 9]. In patients with unfavorable cardiac anatomy, where the heart is close to the thoracic wall, the cardiac doses increase significantly [10, 11].

The critical organs for left-sided breast cancer include the heart and also the lung and contralateral breast. Reports have shown that the long-term risk for developing a second primary breast cancer in the contralateral breast after radiotherapy for the first breast cancer is elevated for women <40 years, inversely related to age at exposure, and dose dependent [12]. The dose response function of lung injury is often gradual and without a clear and consistent “threshold” [13]. Clinically significant symptomatic radiation pneumonitis occurs in approximately 1–5% of patients irradiated for breast cancers [14].

Treatment techniques with beam arrangement based on the tangent angles can have a limited dose to the ipsilateral lung and the contralateral breast but are difficult to generate a concave dose distribution conforming to the breast target. Advanced techniques like intensity-modulated radiation therapy (IMRT), tomotherapy, and volumetric intensity-modulated arc radiation therapy (VMAT) offer the ability to provide a more sophisticated process through the inverse planning procedure, generating a more conformal dose distribution to the breast target, sparing the high dose region to the anterior heart, and improving dose homogeneity [11, 15–18]. Practically, radiation doses delivered from multiple angles, usually with a low dose, spread widely to a large volume. This is an adverse characteristic for left-sided breast cancer treatment. However, by properly designing the treatment plan with the increasing availability of advanced treatment techniques, plans possessing a concave dose distribution with limited doses to the critical heart, lung, and contralateral breast organs should be achievable. The purpose of this study is to develop an optimal treatment plan for early-stage left-sided breast cancer. Hybrid-IMRT (hIMRT) and limited-tomotherapy (lTomo) techniques were designed and compared dosimetrically.

## 2. Materials and Methods

**Figure 1:**
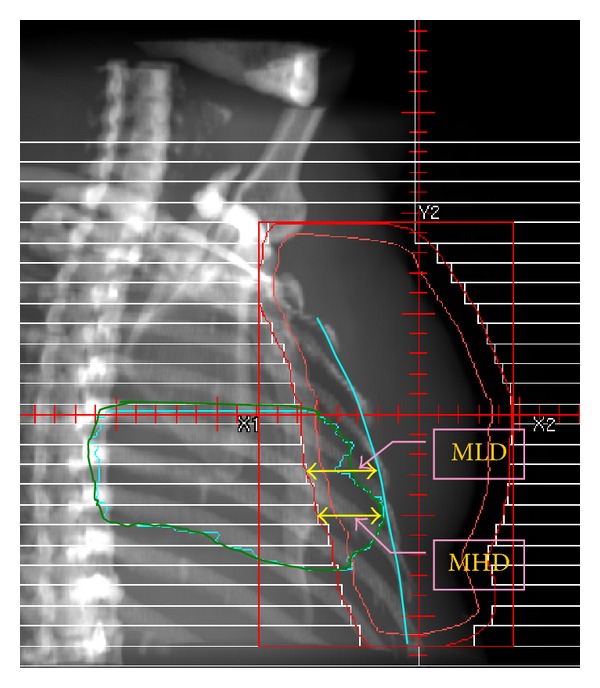
Maximum heart and lung depth measured in the beam's-eye-view of a conventional tangential-field.

**Figure 2:**
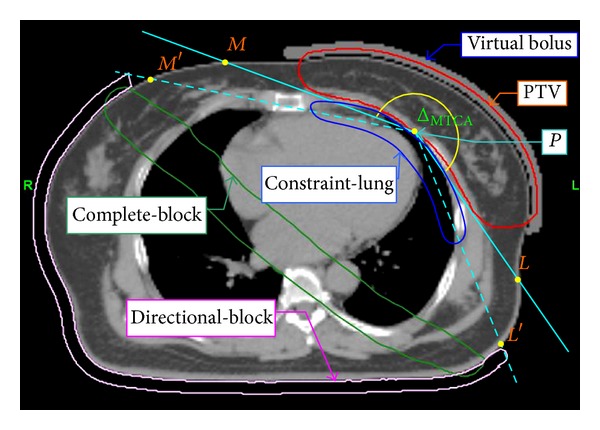
Single CT slice of maximum concave target shape showing the Δ_MTCA_; PTV; 10 mm thickness of virtual bolus; the reference point *P*; lines of *PM*, *PL*, *PM*′, and *PL*′; virtual structures of constraint-lung; complete-block; and directional-block.

Thirty consecutively treated left-sided early-stage breast cancer patients were selected. The maximum heart depth (MHD) [11], the maximum lung depth (MLD) (Figure 1), and the maximum target concave angle (Δ_MTCA_) (Figure 2) were used to express the anatomical conditions of the critical structures and target. Planning CT images were acquired at 3.0 mm slice thicknesses for all patients in the supine position and immobilized on a Vac-Lok bag (CIVCO Medical Instruments, CO, INC., Kalona, IA) with both arms raised above the head. The image set was then transferred to the treatment planning system (Pinnacle3 Version7.6C) for target and organ segmentation. A prescribed dose (*D*_*p*_) of 50.4 Gy in 28 fractions to the planning target volume (PTV) was used.

### 2.1. Target and Critical Structure Segmentation

The clinical target volume (CTV) included the whole left-sided breast. No lymph nodes were included. To account for setup uncertainty and respiratory motion, a margin of 8 mm isotropically was added to the CTV to form the PTV. The part of the PTV that extends into the surrounding air has been called the “flash region” [19, 20]. For adequate “flash region” coverage, a virtual bolus of 10 mm in thickness was used to cover the “flash region.” To avoid dose calculations in a high uncertainty region a PTV-m consisting of the PTV excluding the “flash region” was used for dosimetric evaluation. To account for the tomotherapy treatment planning system implementing a plan without a fluence map, the plan was reoptimized if any change in planning structures was necessary. PTV covered by the virtual bolus was used for optimization and dose calculation for the hIMRT and lTomo plans (Figure 2) [21].

The contralateral breast, ipsilateral and contralateral lung, and heart were contoured for the organs at risk. The MHD, MLD, and Δ_MTCA_ were measured for each patient. The reference point *P* in Figure 2 was assigned as the apex of the concave shape of the PTV on the section with a maximum curvature. A virtual structure (constraint-lung) for dose constraint was contoured for each patient to increase the dose conformity of the PTV and to decrease the dose to the ipsilateral lung and heart (Figure 2).

All plans in this study were optimized with at least 95% of the PTV-m encompassed by the *D*_*p*_. The maximum dose was less than 110% of the *D*_*p*_, and the following dose-volume values were used to set the constraints to reduce the individual critical organ dose according to the anatomy condition of each patient: *V*_5_, *V*_10_, and *V*_20_ to the ipsilateral lung; *V*_3_, *V*_5_, and *V*_10_ to the contralateral lung; *V*_10_, *V*_25_, *V*_35_, and *V*_45_ to the heart, and *V*_5_, *V*_10_ to the contralateral breast.

### 2.2. Hybrid-IMRT Planning Technique

**Figure 3:**
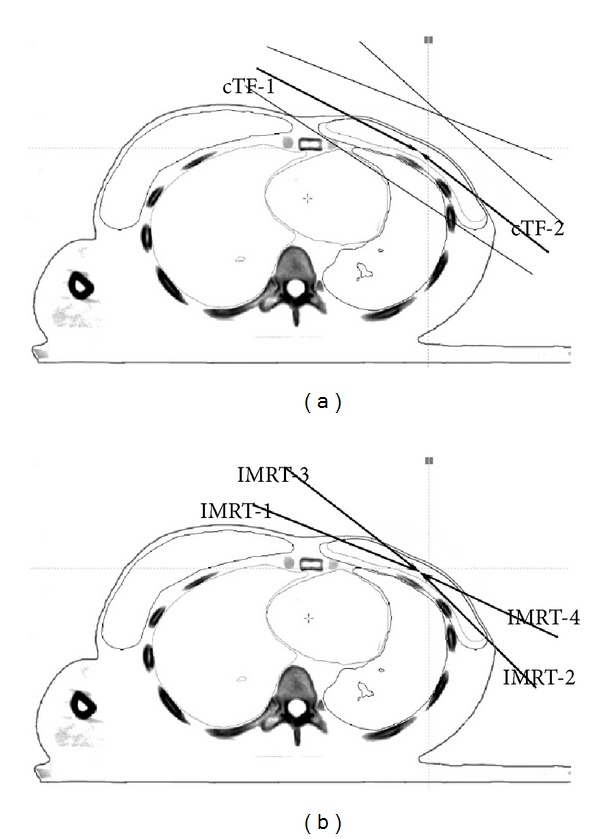
Beam arrangements of the conventional tangential-field plan (a) and the four-field IMRT plan (b). Beam angles of IMRT-1 and IMRT-2 in the 4-F IMRT plan were the same as beam angles of cTF-1 and cTF-2 in the cTF plan, while the others (IMRT-3 and IMRT-4) were 10–15° anterior to the tangential fields.

The hIMRT plans were generated using Pinnacle3 version 7.6C treatment planning system. The hIMRT consisted of a conventional tangential-field plan (cTF) and a four-field IMRT plan (4-F IMRT) using 6 MV energy. The two plans were hybrid using different *D*_*p*_ weightings of 80% and 20%, respectively. Two of the beam angles in the 4-F IMRT plan were the same as the cTF plan while the others were 10–15° anterior to the tangential fields (Figure 3). While processing the 4-F IMRT plan optimization the cTF plan dose distribution was hybrid into the procedure with the optimization type setting as “None.” The contribution of the low weighting 4-F IMRT plan was to increase the dose homogeneity of the hIMRT plan. The maximum number of segments was set to 20 and the minimum segment MUs was set to 4 to effectively perform the 4-F IMRT plan.

### 2.3. Limited-Tomotherapy Planning Technique

DICOM images of each patient with complete target and organ segmentation information were transferred into the tomotherapy Hi-ART planning system (v. 3.2.2.35. TomoTherapy Inc., Madison, WI). The field width, pitch, and modulation factor parameters were assigned to 2.5 cm, 0.287, and 2.8, respectively. Directional blocking was applied to a virtual structure (directional-block), thus closing the beamlets if the blocked structure was proximal to the target to limit the beamlet entrance direction. The ends of the directional-block were at the intersections of the body contour and the *PM*′ and *PL*′ lines. The *PM*′ and *PL*′ lines were approximately 5°–10° posteriorly added from *PM* and *PL* lines, respectively (Figure 2). The judgment of additional angles was a trade-off between critical organ injury and target coverage. To prevent beamlets from entering through the heart and the posterior part of the ipsilateral lung another virtual structure (complete-block) was designed as a rectangular structure with the ends connected to the directional-block to disable beamlets from entering or exiting through this structure (Figure 2). The directional-block and complete-block application dictated that the beamlets could only enter within limited angles from the median and the lateral sides. Critical structures and constraint-lung volume dose constraints were set in the optimization procedure. When optimizing the dose to the constraint-lung was reduced by setting a higher level of dose constraints as a more effective method to reduce the doses to the ipsilateral lung and heart and increase the PTV dose conformity.

### 2.4. Plan Evaluation Parameters

Plans were evaluated based on the homogeneity and conformity for the PTV and the volumes of normal structures irradiated. The PTV homogeneity index (HI) was defined as the difference between the percentage of PTV-m receiving 95% and 107% of the *D*_*p*_ (HI = PTV-m_95%_− PTV-m_107%_). The conformity index (CI) was defined as the fraction of PTV-m that was encompassed by the 95% *D*_*p*_ multiplied by the fraction of the 95% *D*_*p*_ volume that was covered by the PTV-m of the 95% *D*_*p*_ (CI = PTV-m_95%_/PTV-m × PTV-m_95%_/V_95%_). Additionally, the mean and maximum doses of PTV-m (*D*_mean-PTV_,  *D*_max-PVT_) were used as an index of target dose homogeneity.

Normal structure dosimetric evaluation parameters, such as the mean dose, *V*_5_, *V*_10_, and *V*_20_ for lung; mean dose, *V*_10_, *V*_25_, *V*_35_, and *V*_45_ for heart; mean dose, *V*_5_, *V*_10_ for right breast, were used for the plan comparisons. Treatment plans were compared using the paired *t*-test. *P* values of ≤0.05 were considered significant.

## 3. Results

The anatomy conditions of critical structures and targets are shown in Table 1. The mean values and standard deviations (SD) for the MHD, MLD, and Δ_MTCA_ were 1.9 ± 1.0, 2.5 ± 0.6 cm, and 201.5 ± 11.6 degree, respectively. More than 80% of the patients in this study have the MHD larger than 1.0 cm.

**Figure 4:**
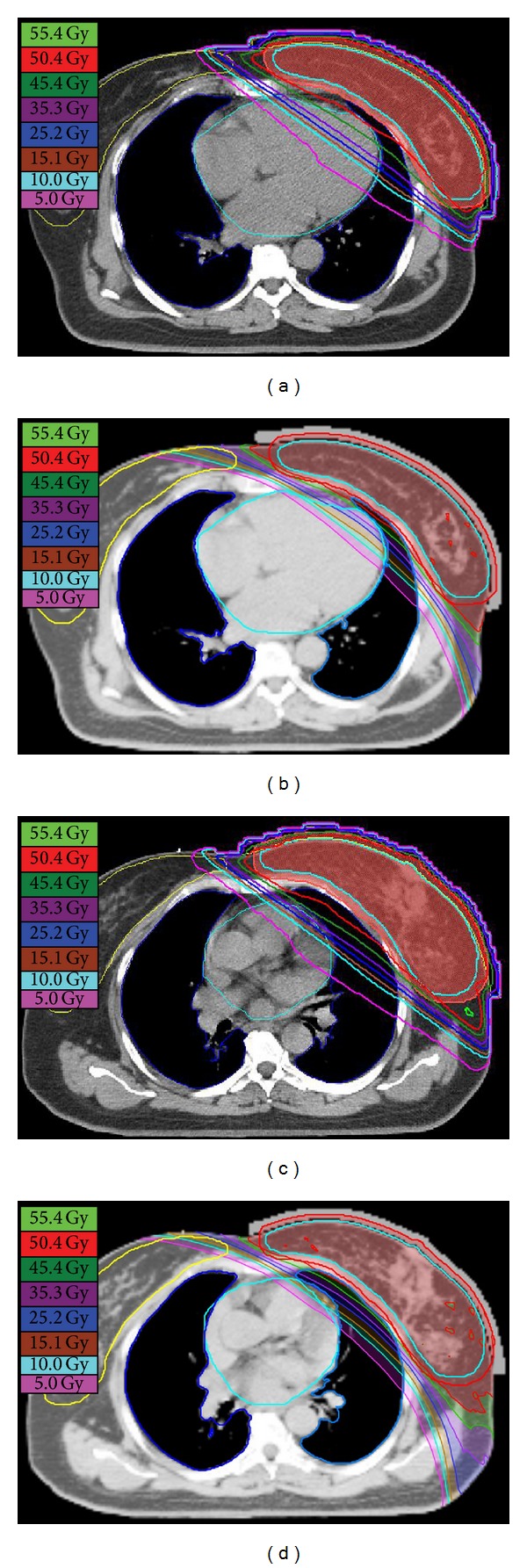
Dose distributions on axial images of heart and lung slices for hIMRT (a, c) and lTomo (b, d) plans from study case number 2.

**Figure 5:**
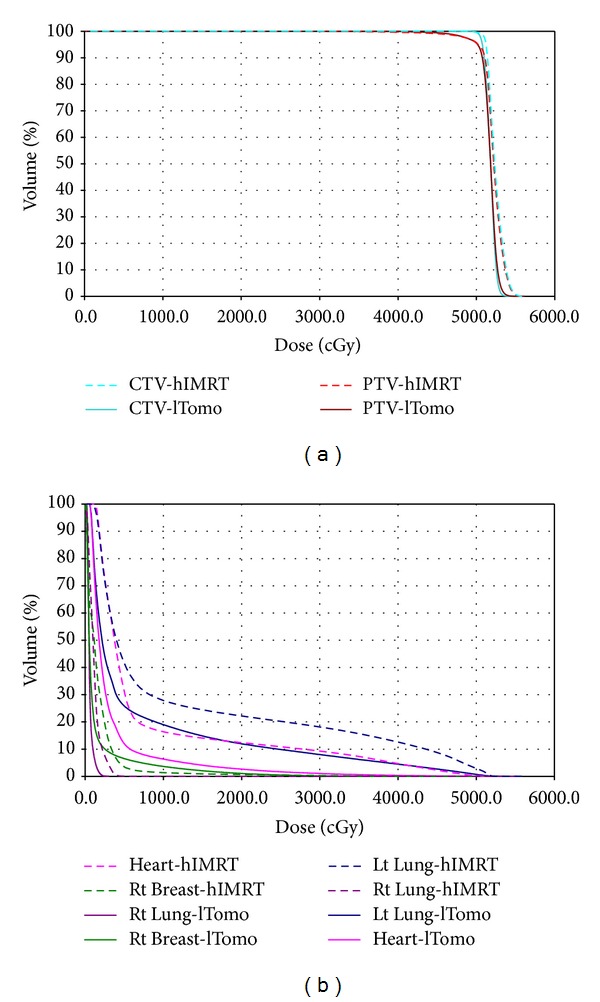
Dose-volume histograms for study case number 2 with hIMRT and lTomo techniques for target (a) and normal structures (b).

The PTV dose coverage parameters and the critical organ dose volume results are displayed in Table 2. Both modalities have similar target coverage. The homogeneity and conformity were improved for lTomo plan with *P* < 0.001 and *P* = 0.006, respectively. Figures 4 and 5 show the dose distributions and DVHs of case number 2 for each modality, respectively. In Figure 4 the lTomo plan, a concave dose distribution along the chest wall, was generated and the doses to the heart and the ipsilateral lung were decreased. The reductions are also shown in DVH (Figure 5(b)). In Table 2, lTomo plan shows a significant dose reduction both in high dose and low dose regions for ipsilateral lung and heart (*P* < 0.001). For contralateral lung, lTomo plan had a lower mean lung dose (*P* < 0.001). For contralateral breast, there was no significant difference between lTomo and hIMRT (*P* = 0.476).

## 4. Discussion

The high incidence rate and long-tern survival rate of female breast cancer make the exposure effect from radiotherapy on the subsequent risk for heart disease and developing a second primary breast cancer an important issue. Both the risks are radiation dose dependent. Traditionally, even advanced techniques widely used in the majority of radiation treatment techniques for breast cancer are still based on the arrangement of tangential beams. The most possible reason is this kind of beam arrangement for the anatomical relationship between the target and critical organs can achieve adequate target coverage and effectively spare the dose to the critical organs. Previous reports published their dosimetric comparison results for left-sided breast irradiation with traditional technique and advanced multibeam treatment techniques [11, 15–17, 22]. A general conclusion from these reports is that advanced treatment techniques can improve target homogeneity and reduce high doses to the heart and lung, but more healthy tissue received low doses.

This study compared the hybrid-IMRT tangential beam based technique and the advanced multibeam limited-tomotherapy technique for reducing the doses to the heart and lung in patients with left-sided early-stage breast cancer. Based on the results of this study, lTomo plan successfully maintained the achievement of previous studies but also significantly reduced the low dose irradiated to other healthy tissues. From Table 2 the average of the mean doses to the heart from the lTomo plans received less than half the dose from the hIMRT plan (*P* < 0.001). The average of the low dose volume (*V*_5_) to the ipsilateral lung from the lTomo plan involved less than 80% of that from the hIMRT plan (*P* < 0.001). Additionally, the lTomo plan also reduced the mean dose to the contralateral lung by approximately 50% (*P* < 0.001). The significant reductions in doses to the healthy tissues using the lTomo plan can improve the treatment quality of left-sided early-stage breast cancer by reducing the subsequent risk for heart disease and radiation pneumonitis.

The tomotherapy Hi-ART planning system offers planner a useful tool to control beamlet entrances by setting a “directional block” or “complete block” structure. Previous studies used this tool to design their plans [11, 15, 17], but only in this study did we geometrically design the limited-arc for beamlet entrance. By contouring the specific virtual structures and setting a “directional block” or “complete block,” dose distributions can be limited to a local region and dose reductions to the ipsilateral or contralateral organs were achieved. Additionally, a constraint-lung virtual structure designed along the posterior chest wall side adjacent to the ipsilateral lung is very helpful to obtaining a concave dose distribution and reducing the high dose region to the heart and lung.

Doses to the normal structures are significantly affected by the anatomical relation between the target and normal structures. MHD and MLD and Δ_MTCA_ were used in this study to support clear information of the anatomical conditions for plan comparisons. Most of the patients in this study presented an unfavorable cardiac anatomy [11].

The disadvantage of the lTomo plan is longer treatment time by approximately 22 minutes. A 5.0 cm field width assigned to the lTomo plan can significantly reduce the treatment time. This is a trade-off between plan quality and time. According to our experience, a comfortable setup and steady fixation are important for keeping patients in the same position during the treatment.

The concept described in this study should be able to apply the VMAT and RapidArc delivery techniques. Further study designed to verify this assumption will be performed in the near future. Additionally, the same concept and method should be applicable for the treatment of extended field breast cancer which includes the internal mammalian lymph nodes.

In conclusion, hIMRT and lTomo provide similar dosimetric target coverage. The concave dose distribution shape conforms to the breast tissue in the lTomo plan, resulting in significant dose reductions to the heart and lung. By properly designing the directionally and complete blocking structure, an lTomo plan was successfully developed to reduce the doses to healthy tissues compared to a conventional tangential-field based plan for early-stage left-sided breast cancer radiotherapy.

## Figures and Tables

**Table 1:** Characteristics of the anatomy conditions of critical structures and targets in this study.

Δ_MTCA_	Number of cases (%)		Mean ± SD

180°–190°	4	13.3%	201.5° ± 11.6°
190°–200°	7	23.3%	
200°–210°	10	33.3%	
>210°	9	30.0%	

MLD (cm)	Number of cases (%)		Mean ± SD

1.0–1.5	2	6.7%	2.5 ± 0.6
1.5–2.0	4	13.3%	
2.0–2.5	5	16.7%	
2.5–3.0	14	46.7%	
>3.0	5	16.7%	

MHD (cm)	Number of cases (%)		Mean ± SD

0.0–1.0	5	16.7%	1.9 ± 1.0
1.0–1.5	8	26.7%	
1.5–2.0	2	6.7%	
2.0–2.5	7	23.3%	
>2.5	8	26.7%	

**Table 2:** Plan evaluation parameters for hIMRT and lTomo plans.

Variable	hIMRT		lTomo		*P* value
	Range	Mean ± SD	Range	Mean ± SD	
PTV					
*D*_mean_ (Gy)	53.08–52.14	52.31 ± 0.09	52.27–51.25	51.68 ± 0.24	<0.001
*D*_max⁡_(Gy)	58.96–54.33	56.39 ± 1.29	58.49–53.10	54.97 ± 1.17	0.014
HI (%)	0.99–0.79	0.92 ± 0.04	1.00–0.97	0.99 ± 0.01	<0.001
CI	0.79–0.55	0.73 ± 0.04	0.82–0.64	0.75 ± 0.04	0.006
Left lung					
*D*_mean_ (Gy)	16.38–6.15	10.03 ± 2.52	9.64–4.37	6.50 ± 1.39	<0.001
*V*_5_ (%)	50.71–21.60	33.14 ± 7.31	40.22–16.63	24.74 ± 5.24	<0.001
*V*_10_ (%)	39.64–14.64	23.54 ± 6.11	26.65–10.92	16.70 ± 4.07	<0.001
*V*_20_ (%)	32.90–9.56	18.64 ± 5.61	17.44–2.50	10.70 ± 3.42	<0.001
Right lung					
*D*_mean_ (Gy)	1.30–0.38	0.94 ± 0.25	1.54–0.27	0.51 ± 0.23	<0.001
Heart					
*D*_mean_ (Gy)	11.12–1.77	6.08 ± 2.74	5.36–0.85	2.76 ± 1.21	<0.001
*V*_10_ (%)	28.56–0.29	12.93 ± 8.02	13.00–0.00	5.14 ± 3.99	<0.001
*V*_25_ (%)	19.32–0.15	8.04 ± 5.64	7.69–0.00	1.80 ± 1.76	<0.001
*V*_35_ (%)	13.94–0.01	5.20 ± 4.11	3.64–0.00	0.82 ± 1.07	<0.001
Right breast					
*D*_mean_ (Gy)	3.78–0.43	1.32 ± 0.78	3.23–0.38	1.33 ± 0.66	0.476
*V*_5_ (%)	12.00–0.02	3.31 ± 3.78	13.98–0.01	5.70 ± 3.66	<0.001
*V*_10_ (%)	7.50–0.00	1.72 ± 2.28	9.77–0.03	2.99 ± 2.44	0.003
